# Biomolecular condensates: Formation mechanisms, biological functions, and therapeutic targets

**DOI:** 10.1002/mco2.223

**Published:** 2023-02-28

**Authors:** Xin Niu, Lei Zhang, Yuchen Wu, Zhi Zong, Bin Wang, Jisheng Liu, Long Zhang, Fangfang Zhou

**Affiliations:** ^1^ Department of Otolaryngology Head and Neck Surgery The First Affiliated Hospital of Soochow University Suzhou China; ^2^ MOE Laboratory of Biosystems Homeostasis & Protection and Innovation Center for Cell Signaling Network Life Sciences Institute Zhejiang University Hangzhou China; ^3^ Department of Orthopedics The First Affiliated Hospital of Wenzhou Medical University Wenzhou China; ^4^ Department of Clinical Medicine, The First School of Medicine Wenzhou Medical University Wenzhou China; ^5^ Institutes of Biology and Medical Science Soochow University Suzhou China

**Keywords:** biomolecular condensates, novel therapies, phase separation

## Abstract

Biomolecular condensates are cellular structures composed of membraneless assemblies comprising proteins or nucleic acids. The formation of these condensates requires components to change from a state of solubility separation from the surrounding environment by undergoing phase transition and condensation. Over the past decade, it has become widely appreciated that biomolecular condensates are ubiquitous in eukaryotic cells and play a vital role in physiological and pathological processes. These condensates may provide promising targets for the clinic research. Recently, a series of pathological and physiological processes have been found associated with the dysfunction of condensates, and a range of targets and methods have been demonstrated to modulate the formation of these condensates. A more extensive description of biomolecular condensates is urgently needed for the development of novel therapies. In this review, we summarized the current understanding of biomolecular condensates and the molecular mechanisms of their formation. Moreover, we reviewed the functions of condensates and therapeutic targets for diseases. We further highlighted the available regulatory targets and methods, discussed the significance and challenges of targeting these condensates. Reviewing the latest developments in biomolecular condensate research could be essential in translating our current knowledge on the use of condensates for clinical therapeutic strategies.

## Graphical abstract

This review highlighted the importance in elucidating the regulation of biomolecular condensates, which is urgently needed for the development of novel therapies. The characteristics, formation mechanism and functions of biomolecular condensates, and the therapeutic targets for diseases are systematically summarized in this review. The available regulatory targets and methods are also discussed in this review. Moreover, this review depicted the significance and challenges of targeting these condensates.

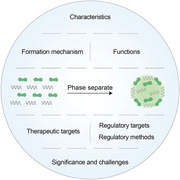

## 1 INTRODUCTION

Biomolecular condensates are membraneless compartments and nonmembrane‐bound bodies, which were first discovered in the 19th century.^1^, ^2^ They exist throughout eukaryotic cells and play an extremely important role in various biological processes.^3^ The formation of these condensates requires their components to undergo a liquid–liquid phase separation or aggregation process, which changes them from a state of dissolution to a state of separation from their surroundings. While the processes of phase separation rely on different elementary steps and ionic strength compared with aggregation, which could form condensates even without phase separation, these two processes are apparently mechanistically connected.^4^ There is a direct link between them in many disease‐associated proteins, that the formation of condensates by phase separation often followed by aggregation to a less dynamic state.^5^

Recent research of condensates has focused on their biological functions with the discovery of properties and molecular mechanisms of their formation and function.^6^ Numerous pathological and physiological processes are associated with dysfunction of the condensates at different scales, which provide promising targets for clinical research on related diseases.^7^ These works provided exciting discoveries and as of late has added to our understanding of the condensates. Therefore, a more extensive description of these condensates is urgently needed for the development of novel therapies.

Here, we have reviewed the current understanding of biomolecular condensates and the molecular mechanisms of their formation and function. Moreover, we summarized the functions of condensates and therapeutic targets for diseases. We further highlight the available regulatory targets and methods, and discussed the significance and challenges of targeting these condensates.

## 2 PROPERTIES OF BIOMOLECULAR CONDENSATES

We propose that there are three properties of biomolecular condensates that determine whether the condensates are formed. Also, the properties obtained by condensates provide new features for cellular components and alter pathological or physiological processes. These properties include: condensates are in liquid‐ or solid‐like state, consist of dynamic components, and present a multiphase immiscible state.

### 2.1 Biomolecular condensates in liquid‐ or solid‐like state

**FIGURE 1:**
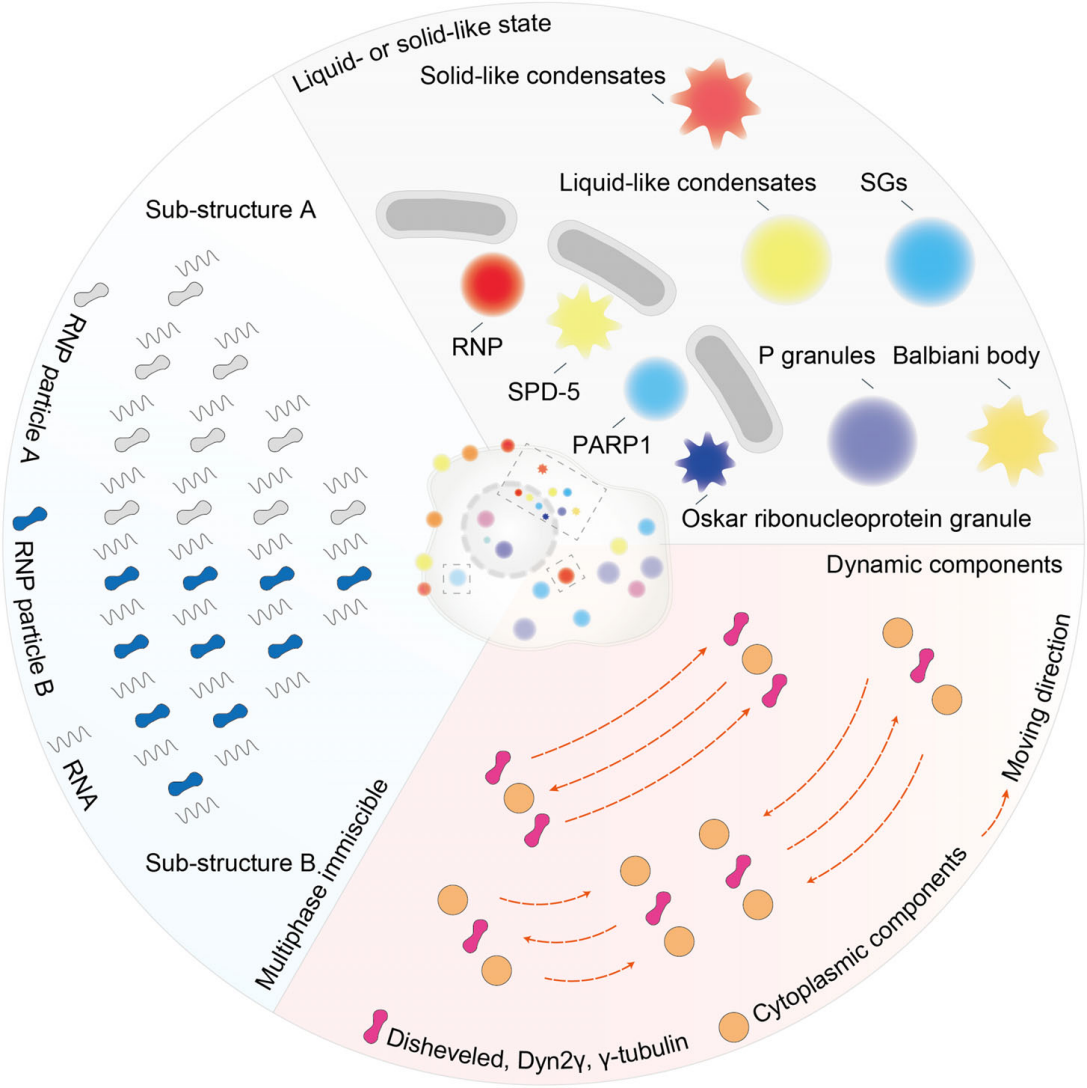
Properties of biomolecular condensates. The three properties of biomolecular condensates. There were a large number of condensates in liquid‐like state at different locations in cells such as SGs, P granules, RNP, and PARP1. The high fluidity of these condensates contributes to their ability to regulate the rate of chemical reactions. There are also a large number of solid‐like condensates present in cells such as Balbiani body, SPD‐5, and Oskar ribonucleoprotein granule. These condensates have less fluidity, but function to protect their contents. The components in the condensates such as Disheveled, Dyn2γ, and γ‐tubulin are able to move freely and dynamically exchange with the surrounding molecules, which support its ability to regulate biochemical processes in cells. The components of the condensates such as P granules, SGs, and paraspeckles are usually not uniformly distributed but in a multiphase immiscible state. RNP, ribonucleoprotein; SPD‐5, spindle‐defective protein 5; PARP1, poly (ADP‐ribose) polymerase 1; SGs, stress granules.

The interaction between proteins or RNAs promotes phase separation and produces micron‐sized droplets (Figure 1).^8^ There are many liquid‐like state and long‐established condensates in cells, such as ribonucleoprotein (RNP) particles, stress granules (SGs), and P granules. RNP particles play a unique role in epigenetic and posttranscriptional regulation.^2^ SGs are formed when cells are stimulated by external pressure to suspend the translation process of mRNA, and they quickly depolymerize to restore normal cell physiological functions when external pressure is relieved. P granules are composed of protein and RNA in the germ cells of *Caenorhabditis elegans*. They flow out of the nucleus under shear force and then drip and fuse into larger droplets.^9^ LAF‐1, the DDX3 RNA helicase in P granules, can also be separated into droplets in vitro.^10^ Moreover, a member of the ADP‐ribosyl transferase family, poly ADP‐ribose (PAR) polymerase 1, recognizes DNA damage sites and synthesizes PAR chains.^11^ PAR attracts fused in sarcoma (FUS) to the DNA damage site and further form droplets rich in damaged DNA.^12^

These condensates are normally in liquid state with high fluidity but can sometimes be more viscous and viscoelastic‐like solids. In RNP particles, inactivation of the RNA helicase results in a transition from liquid to solid state.^13^ The spindle‐defective protein 5 (SPD‐5) is required for centrosome assembly. Recombinant SPD‐5 can form dense droplets in vitro, which are dynamic and liquid at first but then harden.^14^ The Balbiani body in *Xenopus* oocytes is another condensate in solid‐like state. The rigid Balbiani body protects its isolated components from damage and stores macromolecules and organelles for the next generation.^15^ The influx of protons and a pronounced acidification of the cytoplasm also lead to extensive macromolecular assembly of proteins and reduced the fluidity of the cytoplasm, ultimately transforming the cytoplasm into a solid‐like state with stronger mechanical stability.^16^ Moreover, the liquid–solid phase transition is necessary for the formation of the Oskar RNP granule and participate in *Drosophila* embryonic development.^17^ Prion protein (PrP) spontaneously phase‐separates into droplets under physiological conditions and gradually matures into solid‐like β‐rich amyloid with self‐replicating function.^18^ Collectively, biomolecular condensates are either liquid or solid.

### 2.2 Dynamic components in biomolecular condensates

The components in biomolecular condensates maintain a state of free movement and can be dynamically exchanged with surrounding molecules (Figure 1). Fluorescence recovery after photobleaching technology enables the quantitative study of component mobility in these condensates. In P granules, components quickly exchanged with the surrounding cytoplasm or nucleus.^9^ Disheveled is a cytoplasmic protein that acts as a key effector upstream of Wnt signaling pathway, which has a strong propensity to form punctate. It has been reported that there is a continuous material exchange between Disheveled and cytoplasmic components.^19^ Moreover, Dyn2γ or γ‐tubulin complex can be exchanged with cytoplasmic components within a few minutes after photobleaching.^20^

### 2.3 Multiphase immiscible state in biomolecular condensates

Several proteins are contained in biomolecular condensates, while a small number of these proteins is required to promote the formation of droplets in vitro, and most of them likely have a synergistic effect to promote phase separation (Figure 1).^21^, ^22^ The P granules have a heterostructure with a specific composition of RNP particles sequestered in the different region. The assembly of P granules in embryos can be regulated by phosphorylation, suggesting that their substructures have clear molecular specificity.^23^ Jain et al.^24^ reported SGs contain a stable core structure and a dynamic shell. In paraspeckles, AG‐rich RNA and long noncoding RNA *Neat1* are distributed along the boundaries.^25^

Collectively, to understand biomolecular condensates, we must describe as closely as possible the communities of biomolecular which comprise them. Biomolecular condensates contain a variety of substances and present a heterogeneous immiscible state of dense and dilute phases. The accepted criteria for defining a condensate are that it is spherical, fused, and recovered from photobleaching.^26^ Live‐cell fluorescence confocal microscopy and 1,6‐hexanediol are commonly used to characterize the localization or properties of condensates.^27^ However, it should be noted that less evidence is not enough to definitively prove that the structure is a condensate. The fast recovery of photobleaching may be due to the reversible binding of proteins to porous solid structure.^26^ 1,6‐Hexanediol is able to alter the membrane permeability of living cells, which can lead to additional artifacts.^28^

## 3 FORMATION MECHANISMS OF BIOMOLECULAR CONDENSATES

Recent studies have revealed the formation and function mechanisms of biomolecular condensates. These mechanisms empower the components assemble into condensates and further perform various duties. Understanding these mechanisms not only advances our knowledge of biomolecular condensates, but also provides the basis for the research of transformation by using condensates. In this section, we reviewed formation mechanisms of condensates.

### 3.1 The formation of condensates depends on multivalent interactions

**FIGURE 2:**
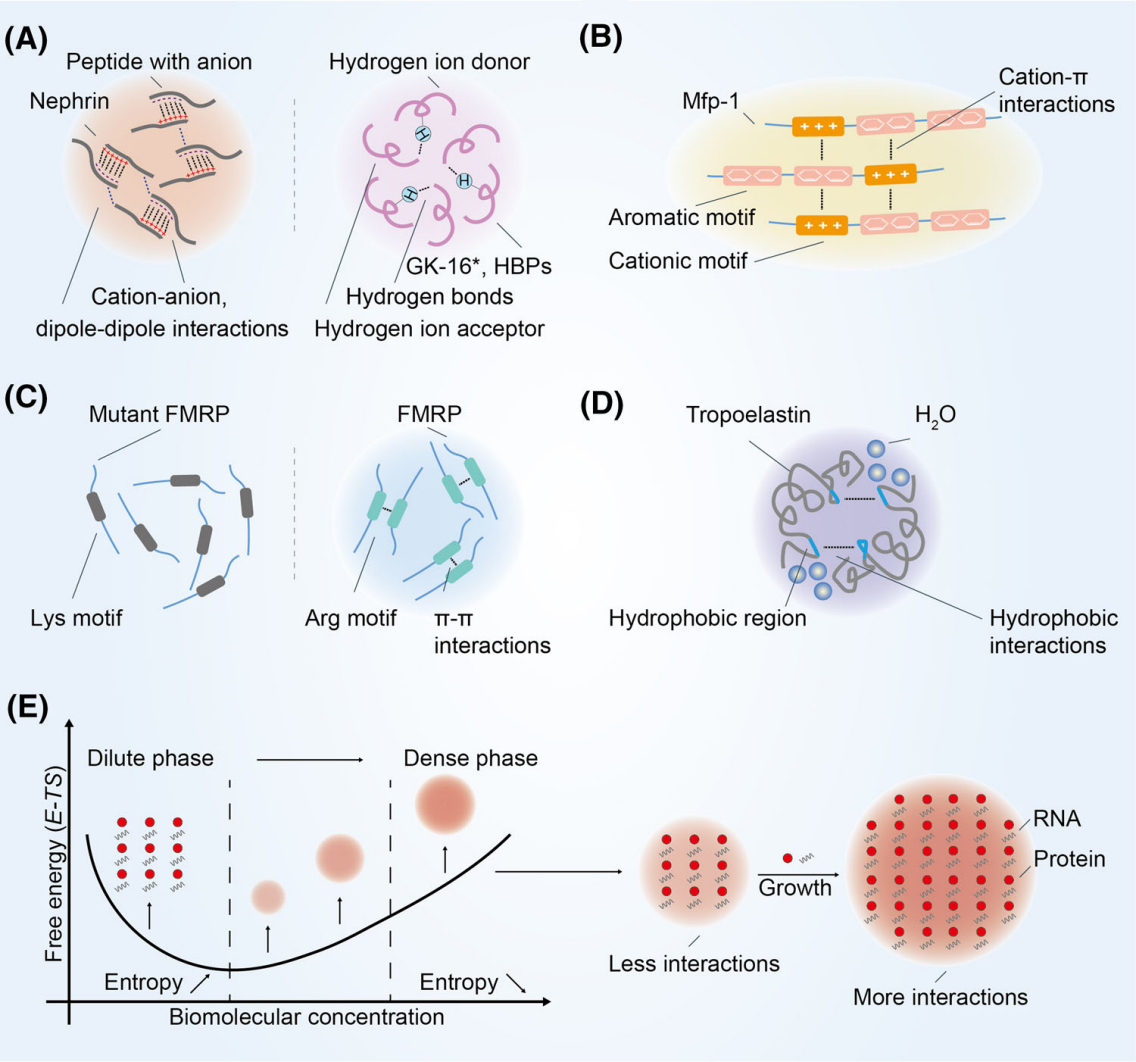
Formation mechanism of biomolecular condensates. (A) Nephrin forms condensates through cation–anion and dipole–dipole interactions. In these interactions, oppositely charged ion and biomoleculars attract each other to promote phase separation. GK‐16* and HBPs form condensates through hydrogen bonds. In these interactions, hydrogen ion donor and acceptor attract each other to promote phase separation. (B) Mfp‐1 forms condensates through cation–π interactions. In these interactions, cations and aromatic amino acid attract each other to promote phase separation. Mfp‐1, mussel foot protein‐1. (C) FMRP forms condensates through π–π interactions. In these interactions, mainly aromatic amino acids such as arginine attract each other to promote phase separation. FMRP, fragile X mental retardation protein. (D) Tropoelastin forms condensates through hydrophobic interactions. In these interactions, biomoleculars with hydrophobic regions attract each other and repel water to further promote phase separation. (E) In the thermodynamic process, condensates are stable when the biomolecular concentration exceeds the saturation concentration. With the increase of biomolecular concentration, the condensates change from dilute phase to dense phase. Small condensates cannot hold onto their proteins as well as larger condensates, which promotes diffusion flux from small to large condensates. *E*, binding energy; *T*, temperature; *S*, entropy.

Recent studies have focused on their biological function with the discovery of their physical properties and production mechanisms. An increasing amount of experimental evidence suggests that multivalent interactions regulates the formation of biomolecular condensates.^3^ There are five main types of multivalent interactions: cation–anion, dipole–dipole, cation–π, π–π, and hydrophobic interactions.^29^ Cation–anion interactions refer to the attractive or repulsive interaction between molecules with cation and anion, and dipole–dipole interactions refer to the attraction between positively charged part and negatively charged part (Figure 2A). The Nephrin intracellular domain is able to assemble with anionic partners to form condensates.^30^ The main component of the nuage or germ granule, Ddx4, needs extremely strong cation–anion and dipole–dipole interaction to facilitate phase separation.^31^ In addition, there is a special type of dipole–dipole interaction known as hydrogen bonding.^32^ Hydrogen bonds allowing polypeptide chain to form secondary protein, which further form tertiary protein through multivalent interactions. It has been reported that a peptide from the primary adhesive protein Mfp‐5, GK‐16^*^, requires dihydroxyphenylalanine and glycine mediated hydrogen bonds for phase separation.^33^ Histidine residues in histidine‐rich squid beak proteins generate condensates by forming hydrogen bonds with tyrosine.^34^ Cation–π interactions are commonly found between amino acid with cation and aromatic amino acid (Figure 2B). In mussel foot protein‐1 residues, cation–π interactions can overcome the repulsion of cation–anion interactions and promoting phase separation of molecules with same charge.^35^ π–π interactions are usually found in aromatic amino acid (Figure 2C). Intriguingly, π–π interactions have also been reported to be present in nonaromatic amino acid such as fragile X mental retardation protein.^36^ Hydrophobic interactions refer to hydrophobic groups that avoid water in close proximity to each other (Figure 2D). Tropoelastin relies on hydrophobic interactions to facilitate phase separation.^37^

### 3.2 Thermodynamic aspects of condensate formation

In the classic thermodynamic context, the formation of condensates is a transient and nonequilibrium process, that is, the initial equilibrium state of the system is changed through appropriate changes in thermodynamic conditions, and the condensates are formed by nucleation, growth, and coarsening.^38^ This is a density transition event that occurs when the concentration of biomoleculars exceeds the saturation concentration, resulting in the formation of a dense phase enriched in biomoleculars, which is relatively depleted by a dilute phase.^39^, ^40^ This concentration threshold also known as the percolation threshold that defines the gel point.^41^ Due to differences in surface curvature, the interaction between biomoleculars on the surface of small condensates less than big condensates further leads to small condensates to lose more easily (Figure 2E). Larger condensates will grow at the expense of smaller condensates.

In addition to the formation of condensates, changes in thermodynamic conditions such as concentration or temperature are also associated with altered size and number of condensates.^29^, ^38^ For example, the size of enhancer condensates depends critically on the concentration of transcription factors (TFs) that bind enhancers.^42^, ^43^ Due to different surface curvature, the number of small to large condensates tends to decrease gradually, and small condensates can grow or shrink rapidly, whereas large condensates takes several days.^40^ The residence time of component reactants is strongly influenced by the size of condensates. Molecular diffusion also depends on the size. It has been reported that the effective mesh size of LAF‐1 condensates is 3–8 nm, which determines the size scale of molecular diffusion and permeability.^44^ Moreover, the initiation of the antiviral immune response may be related to the size of the condensates formed around dsDNA in the cytoplasm.^45^

Together, in the process of condensate formation, peptide with different chemical properties and RNAs with anion could establish various affinity interactions, which enable these component to assemble. Due to entropy‐driven effects, such assembly would inherently reduce the solubility of the molecules, thus promote the formation of condensates. Regulating the formation of biomolecular condensates needs to focus on these multivalent interactions or thermodynamic conditions.

## 4 BIOLOGICAL FUNCTIONS OF BIOMOLECULAR CONDENSATES

### 4.1 The mechanism of biomolecular condensates function

**FIGURE 3:**
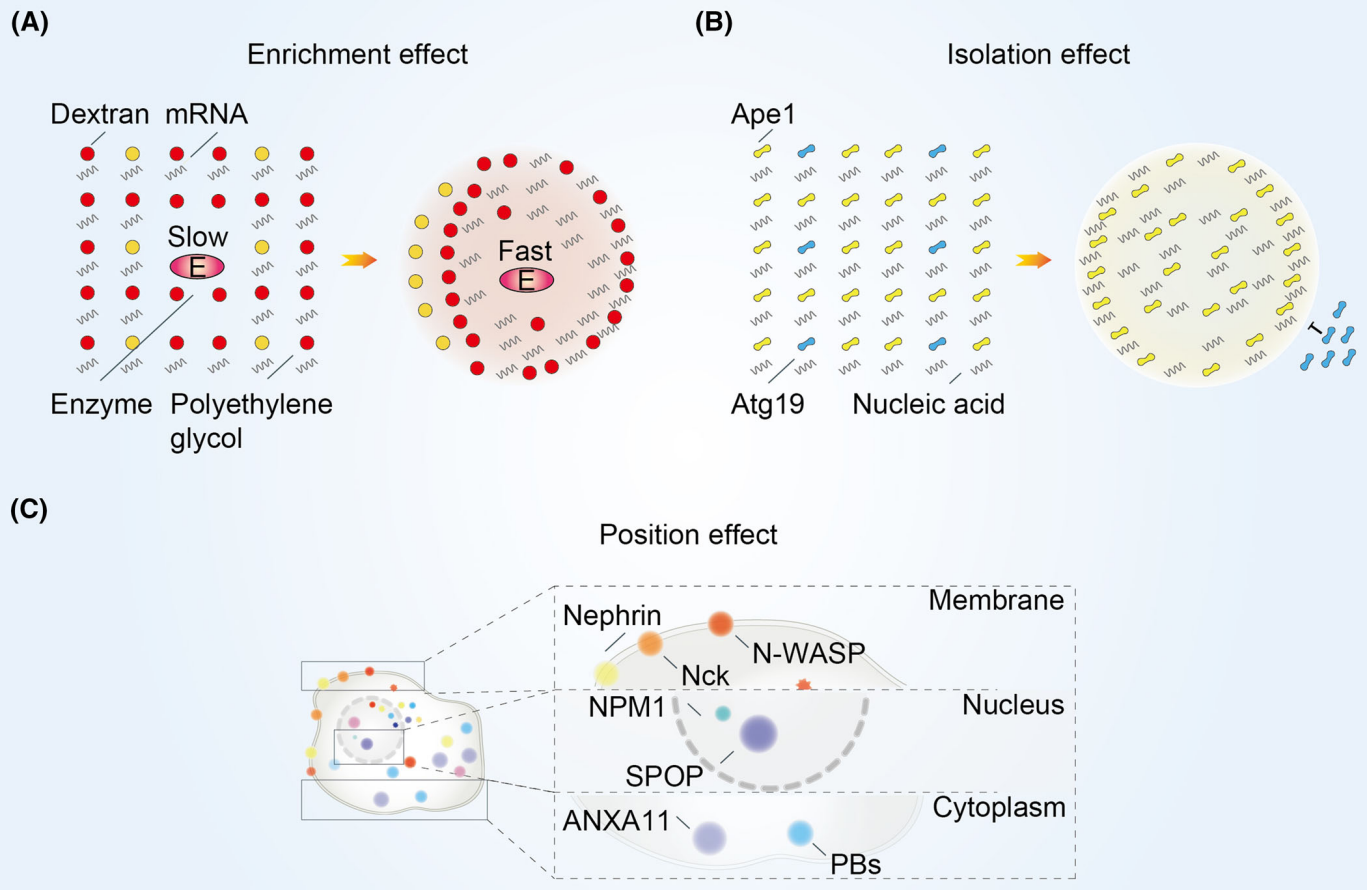
The mechanism of biomolecular condensates function. (A) In the condensates formed by Dextran, Polyethylene glycol and mRNA, the enrichment effect collects most of specific molecules into condensates interior to provide enough reactants for the chemical reaction. (B) In the condensates formed by Ape1 and nucleic acid, isolation effect completely block Atg19 on the outside of the condensates to maintain a stable internal composition. Ape1, aminopeptidase I; Atg19, autophagy‐related protein 19. (C) In localization effect, condensates localize their internal components to specific regions in the cell. Nephrin, Nck and N‐WASP are located in the cell membrane; ANXA11 and PBs are localized in the cytoplasm; NPM1 and SPOP are existed in the nucleus. N‐WASP, neural Wiskott‐Aldrich syndrome protein; NPM1, nucleophosmin 1; SPOP, spotted POZ protein; ANXA11, annexin A11; PBs, P‐bodies.

Biomolecular condensates are able to increase the concentration of their internal components and lead to changes in the chemical reaction rate (Figure 3A). Condensates formed by the polyethylene glycol/dextran aqueous two‐phase system can increase RNA concentration and ribozyme cleavage rates.^46^ Low‐molecular‐weight mononucleotides and cationic peptides facilitate phase separation that further selectively chelate porphyrins, inorganic nanoparticles, and enzymes. This enrichment effect ultimately increases the phosphorylation rate of glucose.^47^ Moreover, biomolecular condensates can also form lateral compartments enriched for particular lipids and proteins on cell membranes.^48^

Biomolecular condensates have isolation effect that enables them to maintain a homeostatic environment (Figure 3B). Heterochromatin components such as nucleosomes and DNA are preferentially distributed in droplets formed by heterochromatin protein 1α.^49^ TIS granules, formed by RNA‐binding protein (RBP) TIS11B, are able to further interweave with the endoplasmic reticulum to form a reticular network and maintain a different physicochemical environment from the cytoplasm.^50^ P granules act as protein size filters in the transport of molecules to the nucleus by excluding proteins larger than 45 kDa.^51^ Moreover, the specific receptor protein for the selective autophagy cargo aminopeptidase I, autophagy‐related protein 19, is localized only on the surface of aminopeptidase I droplets in vitro and in vivo but does not penetrate into the droplets.^52^

Biomolecular condensates also have position effect that allowing them to localize to specific regions of cell (Figure 3C). Nucleophosmin 1 (NPM1) forms condensates in the nucleolus with proteins containing arginine‐rich linear motif and ribosomal RNA.^53^ Spotted POZ protein, a substrate linker for cullin3‐RING ubiquitin ligase, forms condensates at the nuclear speckles.^54^ Besides, P‐bodies (PBs), the condensates formed by mRNA decapping and 5′→3′ degradation reactions occur abnormally, anchored on microtubules and exhibit spatially restricted motion that depends on microtubules.^55^ The N‐terminus of Annexin A11 promotes the formation of membraneless RNA granules, and the C‐terminus interacts with and localizes to lysosomes.^56^ Moreover, Nephrin, Nck, and N‐WASP are able to promote phase separation on lipid bilayer membranes and increase the residence time of N‐WASP and Arp2/3 complexes on the membranes.^57^

### 4.2 Functions of biomolecular condensates

**FIGURE 4:**
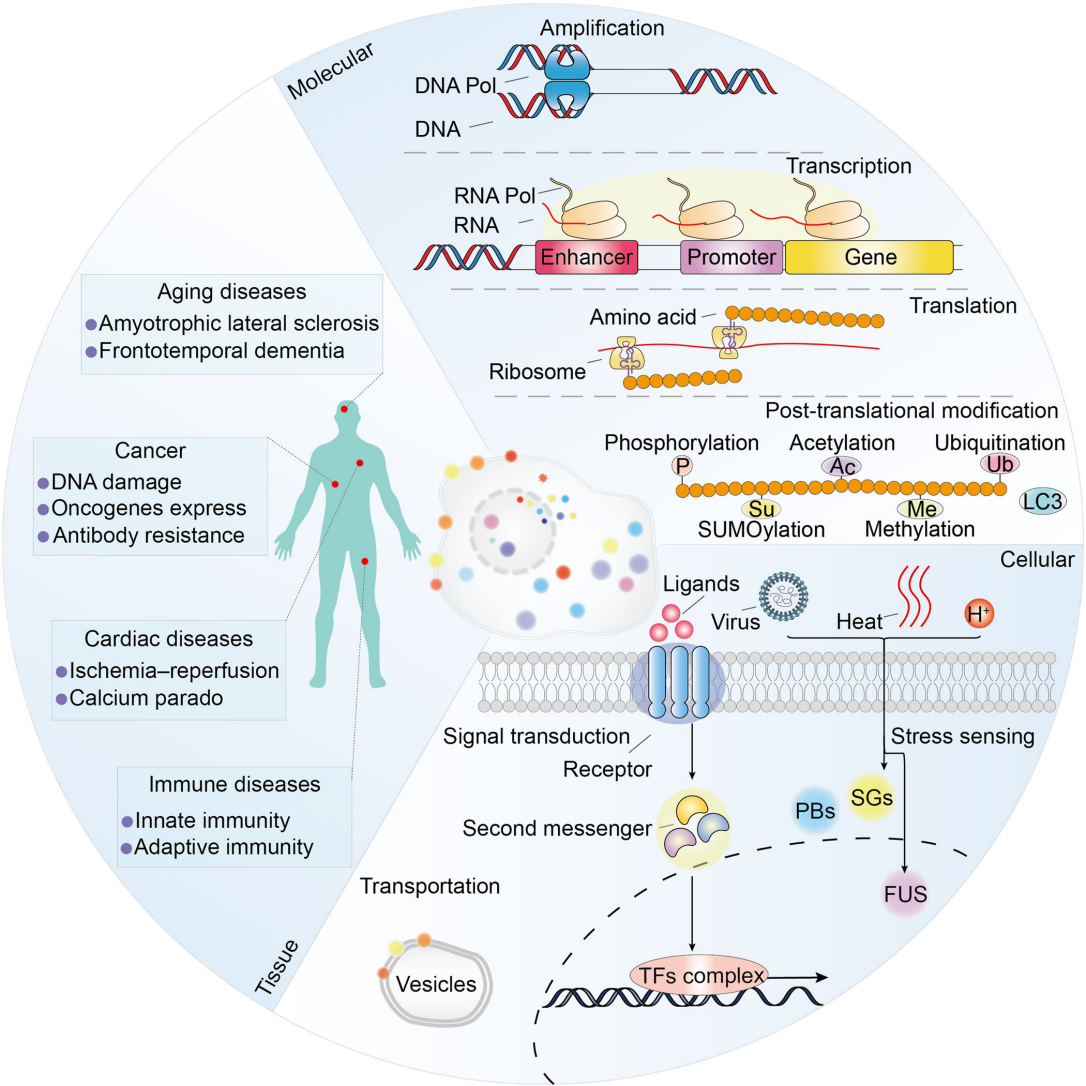
Functions of biomolecular condensates. The functions of biomolecular condensates. At molecular scale, biomolecular condensates are critical for amplification, transcription, translation, and posttranslational modification process such as phosphorylation, acetylation, ubiquitination, SUMOylation, and methylation. At cellular scale, condensates participate in signal transduction, stress sensing, and transportation processes. At tissue scale, condensates involved in DNA damage, oncogenes express, antibody resistance, amyotrophic lateral sclerosis, frontotemporal dementia, ischemia–reperfusion, calcium parado, innate immunity, and adaptive immunity processes. Pol, polymerase; SGs, stress granules; PBs, P‐bodies; FUS, fused in sarcoma.

In recent years, a series of evidences have shown that multifunctional condensates are not only related to physiological processes, but also to pathological processes (Table 1). Given the pivotal role of biomolecular condensates in biological processes, its targeting is promising for clinical research.^7^ In the following section, we summarized the functions of these condensates at three different biological scales including molecular scale, cellular scale, and tissue scale (Figure 4).

**TABLE 1:** Physiological/pathological processes regulated by biomolecular condensates.

Physiological/pathological processes	Condensates	Location	References
Chromatin remodeling	CBX2	Nucleus	^58^
DNA replication	ORC/CDC6/CDT1	Nucleus and cytoplasm	^59^
DNA repair	53BP1	Nucleus	^60^, ^61^
	Autophagosomes	Cytoplasm	^62^
	DAXX	Nucleus and cytoplasm	^63^
	DDX3	Nucleus, cytoplasm, and plasma membrane	^64^
	FUS	Nucleus and cytoplasm	^11^
	NONO	Nucleus	^65^
	PARP	Nucleus	^11^
	RAD52	Nucleus	^66^
	SFPQ	Nucleus and cytoplasm	^67^
	UPS complex	Cytoplasm	^62^
Epigenetic regulation	BRD4	Nucleus	^68^, ^69^
	EWS	Nucleus, cytoplasm, and plasma membrane	^70^
	HP1α	Nucleus	^49^, ^71^
	MALAT1	Nucleus	^72^
	MeCP2	Nucleus	^73^
	NCOA3	Nucleus	^74^
	PRC1	Nucleus, cytoplasm, and plasma membrane	^75^
Telomeres	NONO	Nucleus	^76^
	SFPQ	Nucleus and cytoplasm	^76^
RNA stability	NEAT1	Nucleus	^77^
RNA splicing	HNRNP A1	Nucleus and cytoplasm	^78^
	NONO	Nucleus	^65^
	SFPQ	Nucleus and cytoplasm	^67^
	SRSF2	Nucleus and cytoplasm	^79^
Transcription	CDK7	Nucleus, cytoplasm, and plasma membrane	^80^
	DAXX	Nucleus and cytoplasm	^63^
	DDX3	Nucleus, cytoplasm, and plasma membrane	^81^
	ENL	Nucleus	^82^, ^83^
	EWS	Nucleus, cytoplasm, and plasma membrane	^84^, ^85^
	FCA	Nucleus and cytoplasm	^86^
	FUS	Nucleus and cytoplasm	^87^
	FXR1	Nucleus, cytoplasm, and plasma membrane	^88^
	HSF1	Nucleus	^89^
	MED1	Nucleus	^90^, ^91^, ^92^, ^93^
	MYC	Nucleus	^94^
	NONO	Nucleus	^65^
	NUP98‐HoxA9	Nucleus	^95^
	OCT4	Nucleus and cytoplasm	^94^
	osk	Nucleus and cytoplasm	^17^
	P‐TEFb	Nucleus	^96^
	TAF15	Nucleus and cytoplasm	^97^, ^98^
	TP53	Nucleus and cytoplasm	^99^
	YAP/TAZ	Nucleus and cytoplasm	^100^
	YTHDF1	Cytoplasm	^101^
	YTHDF2	Nucleus and cytoplasm	^102^, ^103^
	YTHDF3	Cytoplasm	^101^, ^104^
	ZNF207	Nucleus and cytoplasm	^105^
Ribosome biosynthesis	NPM1	Nucleus	^106^
Protein degradation	RAD23B	Nucleus and cytoplasm	^107^
	SPOP	Nucleus and cytoplasm	^54^
Transport	Cholesterol	Plasma membrane	^108^
	NPCs	Nucleus	^109^
Signal transduction	CTNNBIP1	Nucleus and cytoplasm	^110^
	DACT3	Cytoplasm	^111^, ^112^
	DAXX	Nucleus and cytoplasm	^63^
	ESR	Nucleus, cytoplasm, and plasma membrane	^94^
	FGF	Nucleus and cytoplasm	^111^
	IKBK	Nucleus and cytoplasm	^113^
	PML	Nucleus and cytoplasm	^114^
	PRKAR1A	Nucleus, cytoplasm, and plasma membrane	^115^
	SOS	Cytoplasm and plasma membrane	^116^
	TJP	Plasma membrane	^117^
	YAP/TAZ	Nucleus and cytoplasm	^118^, ^119^
Immune signaling	CGAS	Nucleus, cytoplasm, and plasma membrane	^45^
	DAXX	Nucleus and cytoplasm	^63^
	IRF3/IRF7	Nucleus and cytoplasm	^120^
	SARS‐CoV NP	Cytoplasm	^121^
	YTHDF1	Cytoplasm	^122^
Stress response	G3BP1	Nucleus and cytoplasm	^123^
	FUS	Nucleus and cytoplasm	^123^, ^124^
	hnRNP A1	Nucleus, cytoplasm, and plasma membrane	^123^, ^125^
	PML	Nucleus and cytoplasm	^114^
	sup35	Cytoplasm	^126^
	TAF15	Nucleus and cytoplasm	^127^
	YTHDF1	Cytoplasm	^128^
	YTHDF2	Nucleus and cytoplasm	^128^
	YTHDF3	Cytoplasm	^128^
Autophagy	ULK1	cytoplasm	^129^

#### 4.2.1 Molecular functions regulated by biomolecular condensates

At the molecular scale, biomolecular condensates are critical for amplification, transcription, translation, and posttranslational modification process. For example, nuclear condensates are critical for the establishment and stabilization of BuGZ functions in the assembly of spindle,^130^ allowing the enrichment of tubulin along microtubules.^105^ Phase transition of chromatin that is induced by global histone deacetylation confers precise movement of chromosomes during cell division.^131^

RNP particles are involved in synthesis of ribosomal RNA, maturation of small nuclear RNA and mRNA.^132^ Mediator promotes phase separation with activation domain of TFs such as Oct4, Sox2, and Nanog, further regulating the transcription process.^94^ Arabidopsis FLL2 promotes the liquid–liquid separation of poly(A) complexes and regulates transcriptional process.^86^ Moreover, condensates formed by RBP FXR1 activate translation of RNA and drive spermiogenesis.^88^ Intriguingly, it has been recently reported that TFs are able to directly activate transcription before phase separation occurs, but the formation of condensates has transcriptional repressive function.^133^, ^134^

Cytoplasmic protein YTHDF induced by heat shock binds to m6A‐modified mRNAs, significantly enhances phase separation, and alerts stability and translation process.^128^ Furthermore, condensates are also involved in UPS and autophagosome process, which provide quality control of protein synthesis.^62^

#### 4.2.2 Cellular processes involved in biomolecular condensates

At the cellular scale, condensates have been found to be closely related to transport and signal transduction. Sphingolipids are commonly found in eukaryotic cells participate in intracellular material transport process and as structural molecules of cell membranes. During the maturation of the Golgi cistern, sphingolipids attract and facilitate phase separation of cholesterol in the bilayer of gall vesicles, further regulating the fusion process of the vesicles.^108^

It was reported that cell surface transmembrane receptors require condensates for signal transduction. In Wnt/β‐catenin signaling pathway, the destruction complex functions by forming condensates.^112^ However, transforming growth factor‐beta induces the expression of DACT1 and forms condensates in the cytoplasm which further inhibits Wnt signaling.^111^ Xue et al.^135^ recently reported basic fibroblast growth factors form condensates with heparan sulphate proteoglycans and activate FGFR signaling pathway. Besides, cytoplasmic scaffolds zonula occludens‐1, zonula occludens‐2, and claudins phase separate to regulate the assembly of tight junctions.^117^

#### 4.2.3 The role of biomolecular condensates in tissue

##### 4.2.3.1 Immunological processes regulated by condensates

Mounting evidence has highlighted that biomolecular condensates are directly relevant to immune signaling and immune modulation.^116^, ^136^, ^137^ On the plasma membrane, immune signaling including T cell receptor (TCR) and B cell receptor (BCR) signaling pathway is regulated by condensates. The main component of the TCR signaling pathway is the discrete TCR microclusters on the membrane. These microclusters consist of a series of proteins including TCR, CD28, PD1, ZAP70, LCK, LAT, PLCγ1, GRB2, and SOS1.^138^, ^139^, ^140^ It has been reported that four proteins in TCR microclusters, LAT, PLCγ1, GRB2, and SOS1, are able to form condensates.^141^, ^142^ These condensates participate in the activation, the baseline level reset after activation of TCR signaling, and the spatial organization of signaling receptors to further influence ligand binding.^140^, ^143^, ^144^ In the BCR signaling pathway, scaffold protein SLP65 and its binding partner CIN85 form condensates in the cytoplasm of resting B cells, and bind to the plasma membrane after BCR activation.^145^ These condensates function to facilitate calcium signaling and downstream signaling pathways.^146^

In the cell, stimulator of interferon genes (STING) pathway, SG pathway, inflammasome pathway, and nuclear factor‐κB (NF‐κB) signaling pathway are regulated by condensates. Cyclic GMP–AMP synthase (cGAS) combines double‐stranded DNA from pathogens or DNA damage, and activate the STING signaling pathway to induce the expression of inflammatory cytokines.^45^, ^147^ Recent reports have shown that cGAS not only binds to DNA and protects it from interpretation, but also forms condensates with RNA and enhance STING signaling pathway when DNA concentration is low.^148^, ^149^, ^150^ STING and its downstream components TBK1 and IRF3 also undergo phase separation process, but they suppress innate immune signaling.^137^, ^151^ Droplets composed of DNA repair proteins such as Rad52 interact with different types of DNA damage‐inducible microtubule filaments, promote the localization of DNA repair proteins to damage sites, and maintain genome stability.^66^ FUS is capable of phase separation at sites of DNA damage.^12^ Moreover, the virus uses condensates to evade immune surveillance. ORF9, ORF52, and VP22 from viruses combined with DNA to form droplets and prevents cGAS activation.^152^, ^153^, ^154^ Nuclear domain 10 forms condensates and restricts the expression of viral genes after viral DNA invades nucleus.^155^ In recent years, SARS‐CoV‐2 has emerged as an urgent threat to global public health, which virus is assembled by the interaction of SARS‐CoV‐2 nucleocapsid protein (SARS2‐NP), viral RNA genome, and M protein.^156^, ^157^ SARS2‐NPs can form condensates that impair the binding of mitochondrial antiviral signaling proteins and IFN response for immune evasion and attract RNA‐dependent RNA polymerase complex to promote virus replication.^121^, ^158^

Under external stress conditions, eukaryotic cells form SGs and PBs, which store large amounts of mRNA and inhibit the translation process of cells.^159^ Recent studies have shown that G3BP1 bind to RNF125 in virus‐induced SGs to promote its degradation.^160^ G3BP1 is also able to directly interact with proteins such as retinoic acid‐inducible gene I and protein kinase R to act as a positive regulator of the innate immune pathway and block protein translation and viral replication.^161^ PrP Sup35 forms condensates under external pressure and specific pH conditions in yeast.^126^ Thermoregulatory protein heat‐shock TF 1 generates condensates and efficiently drives gene transcription of heat‐shock‐proteins to maintain intracellular protein homeostasis.^162^

Inflammasome is composed of sensor proteins, apoptosis‐associated speck‐like protein containing a CARD, and Caspase 1, which regulates the response to viral invasion through cytokine secretion and gasdermin D mediated pyroptosis.^163^ Shen et al.^164^ found that NLRP6 inflammasome can form condensates with double‐stranded RNA and is efficiently triggered by lipoteichoic acid.

The TFs of the NF‐κB signaling pathway include p65, RelB, c‐Rel, NF‐κB1, and NF‐κB2, which regulate numerous inflammatory mediators, chemokines, and cytokines to activate immune response.^165^ In cells infected with respiratory syncytial virus, p65 subunit, MAVS, and MDA5 tend to form condensates in the vicinity of the nucleus, which prevent them from being transferred to the nucleus to activate transcription of antiviral genes.^166^ In addition, the IκB kinase complex is responsible for activating the NF‐κB signaling pathway. NF‐κB essential modulator is a subunit of the IκB kinase complex, which can form condensates by combining ubiquitin‐binding domain, zinc finger, and K63‐linked or linear polyubiquitin chains, and further activate NF‐κB signaling.^113^

##### 4.2.3.2 Other processes regulated by condensates

At the tissue scale, a range of diseases including cancer, aging, and cardiac diseases are associated with abnormality condensates. Alternative lengthening of telomeres‐associated promyelocytic leukemia nuclear bodies forms condensates to maintain telomere length in response to DNA damage.^167^ NUP98–HOXA9 fusion protein forms puncta and regulates oncogene transcription in phase separation‐dependent process.^168^ SS18 has oncogenic activity by forming condensates in synovial sarcoma.^169^ Glutamine in tumor microenvironment competitively inhibits mitochondrial fission in macrophages, maintains phase separation of WIP/WASP, and results in clinical antibody resistance.^170^ Protein arginine methyltransferases form condensates, and its inhibitor have therapeutic effects in cancer and aging diseases.^171^ Moreover, mislocalization of RBPs outside the nucleus such as FUS, TAF15, hnRNP A1, hnRNP A2, and TDP‐43 leads to amyotrophic lateral sclerosis and frontotemporal dementia.^172^ Furthermore, phosphatidylserine and phosphatidylethanolamine are present in the cytoplasmic globules and phase separate in the presence of calcium ions. These condensates are associated with structural changes in heart muscle cells following ischemia–reperfusion and calcium paradox.^173^

In conclusion, biomolecular condensates play important roles at the molecular, cellular, and tissue scales. Therapies targeting various condensate diseases can be envisaged.^174^ Biomolecular condensates provide emerging explanations for a number of previous biological processes. The synergy between biotechnology, pharmaceutical industry, and expertise from disparate fields is the key to the clinical application of these condensates.

## 5 BIOMOLECULAR CONDENSATES SERVE AS THERAPEUTIC TARGETS FOR DISEASES

**FIGURE 5:**
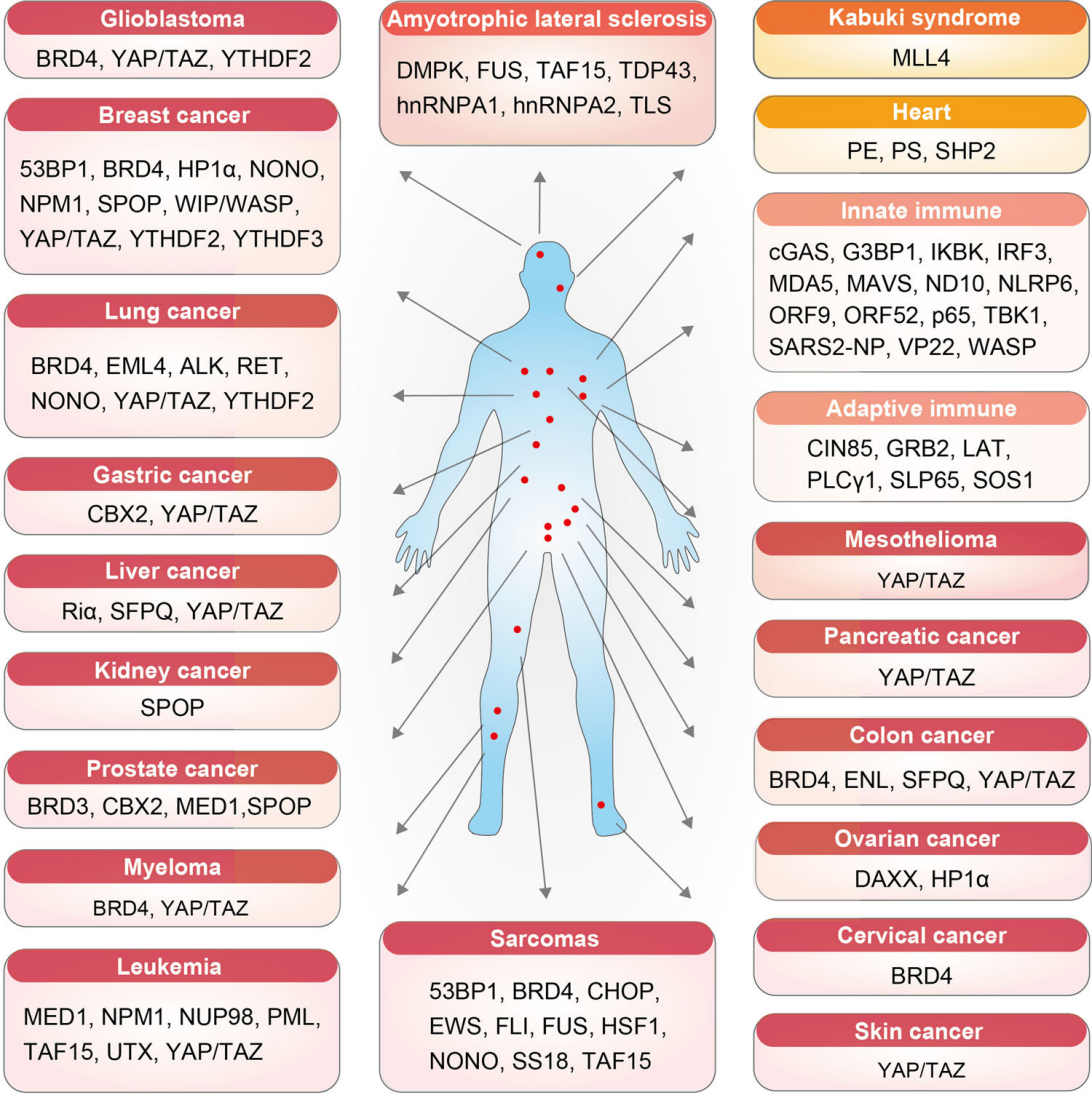
Therapeutic condensates in different diseases. The therapeutic condensates in different diseases. These diseases include: glioblastoma, breast cancer, lung cancer, gastric cancer, liver cancer, kidney cancer, prostate cancer, myeloma, leukemia, sarcomas, skin cancer, cervical cancer, ovarian cancer, colon cancer, pancreatic cancer, and mesothelioma; amyotrophic lateral sclerosis; kabuki syndrome; heart diseases; innate and adaptive immune diseases.

The extensive biological effects of biomolecular condensates make them potential therapeutic targets for diseases. An increasing number of studies indicates that condensates are involved in various disease processes (Figure 5 and Table 2). Here, we the reviewed diseases for which condensates can be used as therapeutic targets. These diseases include cancers, amyotrophic lateral sclerosis, kabuki syndrome, and heart and immune disease.

**TABLE 2:** Therapeutic condensates in different diseases.

Diseases	Therapeutic condensates	Mechanisms	References
Glioblastoma	BRD4	BRD4 participate in super enhancers organization and oncogenes expression regulation.	^175^
	YAP/TAZ	YAP/TAZ activates target genes and is directly involved in the control of S‐phase entry and mitosis.	^176^
	YTHDF2	YTHDF2 directly bound to the m6A modification sites of LHPP and NKX3‐1 to mediate the mRNA degradation.	^177^, ^178^
Amyotrophic lateral sclerosis	DMPK	The accumulation of repeat containing transcripts into aberrant foci in the nucleus.	^179^
	FUS/TLS	The phase changes into an insoluble fibrous hydrogel that is different from conventional amyloid.	^12^
	TAF15/hnRNP A1/hnRNP A2	Their nuclear input is disrupted, as shown by disrupted RNA metabolism and alternative splicing.	^171^, ^172^
	TDP‐43	Condensates were required for efficient TDP‐43 assembly on subsets of RNA‐binding regions.	^180^
Kabuki syndrome	MLL4	MLL4 loss of function impaired Polycomb‐dependent chromatin compartmentalization and alerted nuclear architecture.	^181^
Heart	PE/PS	In a mixture of PE and PS, calcium is capable of inducing fusion events that lead to irreversible destruction of the sarcolemma.	^173^
	SHP2	Disease‐associated SHP2 mutants can recruit and activate wild‐type SHP2 in condensates to promote MAPK activation.	^182^
Innate immunity	cGAS	cGAS condensates can effectively sense immunostimulatory DNA.	^148^, ^150^
	G3BP1	G3BP1 condensates promote innate immune responses and integrates cellular stress responses and innate immunity.	^161^
	IKBK	polyUb activates IKK and NF‐κB signaling by promoting the phase separation of IKBK.	^113^, ^183^
	IRF3/IRF7	Condensates stimulating type I IFN (IFN‐I) expression.	^120^
	ND10	ND10 converge at viral DNA and place restrictions on viral gene expression.	^155^
	NLRP6	NLRP6 condensates have antimicrobial immunity.	^164^
	ORF9	ORF9 directly interacts with cGAS and forms condensates with DNA.	^153^
	ORF52/VP22	ORF52/VP22 proteins accumulate and effectively destroy the preformed cGAS‐DNA condensates.	^154^
	p65/MAVS/MDA5	RSV viral RNA replication takes place in these structures.	^166^
	SARS2‐NP	Condensates recruit RNA‐dependent RNA polymerase complex of SARS‐CoV‐2 for efficient transcription of viral RNA.	^158^
	STING/TBK1	Condensates constrained STING and TBK1 to prevent innate immunity from overactivation.	^137^
	WASP	Loss of WASP condensates promoters leads to aberrant epigenetic activation.	^184^
Adaptive immunity	GRB2/SOS1	SOS1 acts both as a RasGEF and as a scaffold to nucleate GRB2‐dependent adaptor oligomerization.	^141^
	LAT/PLCγ1	PLCγ1 protects LAT from dephosphorylation by the phosphatase CD45 and promotes LAT‐dependent ERK activation and SLP76 phosphorylation.	^142^
	SLP65/CIN85	SLP65/CIN85 complex is responsible for Ca^2+^ and NF‐κB responses.	^146^
Mesothelioma	YAP/TAZ	YAP/TAZ activates target genes and is directly involved in the control of S‐phase entry and mitosis.	^185^
Pancreatic cancer	YAP/TAZ	YAP/TAZ activates target genes and is directly involved in the control of S‐phase entry and mitosis.	^176^
Colon cancer	BRD4	BRD4 participates in super enhancers organization and oncogenes expression regulation.	^186^
	ENL	Condensates are necessary for rapid transcriptional induction.	^83^
	SFPQ	SFPQ interacts with lncRNA‐422 to activate downstream pathways.	^187^
	YAP/TAZ	YAP/TAZ activates target genes and is directly involved in the control of S‐phase entry and mitosis.	^188^
Ovarian cancer	DAXX	DAXX overexpression enhanced the proliferation, colony formation, and migration of ovarian cancer cells.	^189^
	HP1α	HP1α promotes abnormal growth and the potential for neoplasia.	^190^
Cervical cancer	BRD4	BRD4 participates in super enhancers organization and oncogenes expression regulation.	^191^, ^192^
Skin cancer	YAP/TAZ	YAP/TAZ activates target genes and is directly involved in the control of S‐phase entry and mitosis.	^100^
Sarcomas	53BP1	Condensates cause an elevated p53 response and impair cell survival in cancer cells.	^193^
	BRD4/CHOP/FUS	BRD4, CHOP, and FUS form condensates and act as super enhancers.	^194^
	EWS/FLI	EWS and FLI forming local high‐concentration hubs of TFs.	^84^, ^85^
	HSF1	HSF1 is a chaperone transcriptional regulator that is fundamental to cell survival or death.	^89^, ^195^
	SS18	Condensates contribute to the oncogenic activity of SS18‐SSX in synovial sarcomas.	^169^
	TAF15	Condensates promote transcription of abnormal genes and contribute to their oncogenic transformation in sarcomas.	^196^
Myeloma	BRD4	BRD4 participates in super enhancers organization and oncogenes expression regulation.	^197^, ^198^
	YAP/TAZ	YAP/TAZ activates target genes and is directly involved in the control of S‐phase entry and mitosis.	^199^, ^200^
Prostate cancer	BRD4	BRD4 participates in super enhancers organization and oncogenes expression regulation.	^201^
	CBX2	CBX2 promoted the proliferation, invasion and migration of cells by activating the YAP/β‐catenin pathway.	^202^
	MED1	MED1 is essential for androgen receptor mediated transcription.	^203^
	SPOP	Disease‐associated SPOP mutations that lead to the accumulation of proto‐oncogenic proteins.	^54^
Kidney cancer	SPOP	Condensation fails to promote the degradation of androgen receptor located in the nucleus, leading to activation of cancer progression.	^204^
Leukemia	MED1	MED1 plays an important role in gene‐specific transcriptional activation and maintenance of leukemia.	^92^
	NPM1	NPM1‐mutated AML is a WHO classification for lympho‐hematopoietic tumors.	^205^
	NUP98‐HoxA9	NUP98–HoxA9 results in transcriptional activation of leukemogenic genes.	^95^, ^168^
	PML	Mutations in the PML/RARA arsenic‐binding site lead to drug resistance in patients.	^206^
	TAF15	Condensates promote transcription of abnormal genes and contribute to their oncogenic transformation in leukemia.	^196^
	UTX	Loss of IDR in UTX is responsible for abolishing tumor suppression.	^207^
	YAP/TAZ	YAP/TAZ activates target genes and is directly involved in the control of S‐phase entry and mitosis.	^208^
Liver cancer	Riα	RIα condensates induce aberrant cAMP signaling.	^115^
	SFPQ	SFPQ leads to cisplatin resistance in liver cancer.	^209^
	YAP/TAZ	YAP/TAZ activates target genes and is directly involved in the control of S‐phase entry and mitosis.	^210^
Gastric cancer	CBX2	CBX2 promoted the proliferation, invasion, and migration of cells by activating the YAP/β‐catenin pathway.	^211^
	YAP/TAZ	YAP/TAZ activates target genes and is directly involved in the control of S‐phase entry and mitosis.	^176^
Lung cancer	BRD4	BRD4 participates in super enhancers organization and oncogenes expression regulation.	^198^
	EML4/ALK/RET	Condensates locally concentrate the RAS activating complex and activate RAS in a lipid membrane‐independent manner.	^212^
	NONO	NONO is a key regulator for cancer proliferation through the pre‐mRNA splicing of cell proliferation‐related genes.	^65^
	YAP/TAZ	YAP/TAZ activates target genes and is directly involved in the control of S‐phase entry and mitosis.	^176^
	YTHDF2	SUMOylation of YTHDF2 increases its binding affinity of m6A‐modified mRNAs and results in deregulated gene expressions.	^103^
Breast cancer	53BP1	Condensates cause an elevated p53 response and impair cell survival in cancer cells.	^193^
	AKAP95	Condensates regulating gene expression and supporting tumorigenesis.	^213^
	BRD4	BRD4 participates in super enhancers organization and oncogenes expression regulation.	^214^
	HP1α	HP1α promotes abnormal growth and the potential for neoplasia.	^215^
	NONO	NONO is a key regulator for cancer proliferation through the pre‐mRNA splicing of cell proliferation‐related genes.	^65^
	NPM1	NPM1 binds to PD‐L1 promoter specifically in TNBC cells and activates PD‐L1 transcription, thus inhibiting T cell activity.	^216^
	SPOP	Disease‐associated SPOP mutations that lead to the accumulation of proto‐oncogenic proteins.	^54^
	WIP/WASP	Condensates prevent protein kinase C‐θ to phosphorylate WIP during phagocytosis.	^170^
	YAP/TAZ	YAP/TAZ activates target genes and is directly involved in the control of S‐phase entry and mitosis.	^100^, ^217^
	YTHDF2	YTHDF2 interacts with mRNAs encoding proteins in the MAPK pathway and increases global translation rates.	^102^
	YTHDF3	YTHDF3 enhances the translation of transcripts associated with tumor metastasis.	^104^

### 5.1 Condensates are therapeutic targets for a variety of cancers

In breast cancer, a large number of condensates with regulatory effects have been found. p53‐binding protein 1 is capable of phase separation in chromatin, resulting in an elevated p53 response that impairs cancer cell survival.^193^ AKAP95, a nuclear protein that regulates transcription and RNA splicing, supports tumorigenesis by forming condensates and regulating gene expression.^213^ HP1α can form condensates, which has a regulatory effect on the growth of breast cancer.^49^, ^215^ The condensates of RBP non‐POU domain‐containing octamer binding (NONO) regulate tumor cell proliferation by binding to mRNA of proliferation‐related genes.^218^ Mutations of speckle‐type pox virus and zinc finger protein alter the formation of condensates and promote breast cancer progression.^54^ The condensate NPM1 specifically binds to the PD‐L1 promoter in breast cells and activates PD‐L1 transcription, further inhibiting T cell activity in vitro and in vivo.^106^, ^216^ WIP/WASP produces condensates that prevent the protein kinase C‐θ from phosphorylating WIP and reduce efficacy of promising therapeutic antibodies.^170^ Furthermore, YTHDF3 increases breast cancer metastasis by enhancing the translation of m6a‐enriched transcripts of ST6GALNAC5, GJA1, and EGFR.^104^

Lung cancer or non‐small cell lung cancer includes lung adenocarcinoma, lung squamous cell carcinoma, and large cell lung cancer. BRD4 inhibitor JQ1 inhibits proliferation of non‐small cell lung cancer cell line subsets by inhibiting FOSL1 expression.^198^ EML4/ALK/RET activates RAS signaling pathway in lung cancer cells through the formation of condensates.^212^ *SKP2* RNAs, the target of NONO, are highly expressed in small cell and non‐small cell lung cancer. Inhibition of *SKP2* can induce apoptosis of tumor cells and inhibit the cell invasion.^65^ YAP and TAZ are widely activated in human malignancies and are essential for the initiation or growth of most solid tumors, which induces cancer cell proliferation, drug resistance, and metastasis. It has been reported that increased expression or nuclear localization of YAP or TAZ was associated with higher histological grade, advanced TNM, and lymph‐node metastasis of lung cancer.^176^ In addition, SUMOylation of YTHDF2 significantly increases its binding affinity to m6A‐modified mRNA, subsequently leading to dysregulation of gene expression in lung cancer progression.^103^

Liver cancer includes hepatocellular carcinoma, cholangiocarcinomas, and hepatoblastoma. cAMP‐dependent protein kinase type I regulatory subunit, RIα, can form condensates rich in cAMP and PKA activity. Loss of RIα in normal cells increases cell proliferation and induces cell transformation. PKA fusion oncoprotein associated with liver cancer can effectively block the formation of condensates and improve the abnormal cAMP signaling pathway.^115^ An RNA‐ and DNA‐binding protein, splicing factor proline‐ and glutamine‐rich, can induce cisplatin resistance in liver cancer cells.^209^ Recent studies have shown that splicing factor proline‐ and glutamine‐rich is essential for DNA repair and paraspeckle formation and is capable of forming condensates.^219^ Increased expression of YAP or TAZ was also associated with poor prognosis of liver cancer.^210^

In leukemia, condensates of MED1 play an important role in the E2A–PBX1‐driven gene‐specific transcriptional activation and the maintenance of leukemia.^92^ Genetic abnormalities caused by NPM1 mutations often occur in human acute myeloid leukemia, which accounts for about one‐third of all cases.^205^ The condensates of NUP98–HOXA9, which regulate transcriptional activity and transform hematopoietic cells, are critical for the development of leukemia.^168^ Nuclear bodies PML play a key role in the treatment of acute promyelocytic leukemia and are druggable.^206^ FUS/EWS/TAF15 fusion oncoproteins were able to promote abnormal gene transcription by forming condensates and contribute to their oncogenic transformation ability in leukemia.^196^ UTX is an important tumor suppressor that encodes the histone H3K27 demethylase and regulates genome‐wide histone modification and higher‐order chromatin interactions by forming condensates.^207^ Moreover, increasing the level of YAP1 in malignant hematologic diseases can restore the apoptosis induced by nuclear ABL1 kinase and alleviate the disease process.^208^

Gliomas include astrocytoma, oligodendroglioma, oligoastrocytoma, and glioblastoma multiforme. BRD4, YAP/TAZ, and YTHDF2 have regulatory effects on glioblastoma. BRD4, YAP/TAZ form condensates and participate in the assembly of super enhancers to further regulate cellular gene transcription.^90^, ^220^ Inhibition of BRD4 can inhibit the proliferation of glioma cells and promote the apoptosis of tumor cells, and knockout of YAP/TAZ can prevent tumor formation in SCID mice injected with primary cancer cell lines in situ.^175^, ^176^ YTHDF2 stabilizes MYC and VEGFA transcription in glioma cells in an m6a‐dependent manner and enhances tumor activity.^178^, ^221^

In clinical prostate cancer tumor samples, BRD4 protein levels were inversely associated with tumor response after radiation therapy.^201^ The condensates of CBX2 organize the Polycomb group, which inhibits major regulators of development and differentiation by organizing chromatin structure.^58^ The use of inhibitors of CBX2 in the treatment of prostate cancer is highly desirable.^202^ CDK7‐specific inhibitor THZ1 also inhibits prostate tumor growth by blocking MED1 corecruitment genome‐wide and reverses the drug‐resistant phenotype caused by hyperphosphorylated MED1.^203^ Furthermore, speckle‐type pox virus and zinc finger protein mutations contribute to prostate cancer by accumulating proto‐oncogene proteins that interfere with phase separation and colocalization in membraneless organelles.^54^

In Ewing's sarcoma tumors, condensates formed by EWS and FLI are functionally associated with transactivation capacity and carcinogenic potential of cells.^84^ BRG1/BRM‐associated factor complexes can also be recruited by EWS–FLI1 fusion proteins into tumor‐specific enhancers and contribute to target gene activation.^85^ Condensates of FUS/EWS/TAF15 can also promote the abnormal gene transcription and the oncogenic transformation ability in sarcoma.^196^ Furthermore, the FUS–CHOP fusion protein can also form condensates with BRD4 and caused myxoid liposarcoma.^194^ Besides, heat‐shock factor 1 is a transcriptional regulator of chaperone and is capable of forming condensates. Inhibiting the formation of these condensates can promote the activity of heat shock factor 1 and cell survival and reduce the incidence of sarcoma.^89^, ^195^ The condensates of SS18 recruit BRG1, which can be used as a marker for synovial sarcoma.^169^ p53‐binding protein 1 is associated with various DNA repair or cell cycle factors and is involved in the cell's response to DNA double‐strand breaks.^193^

Gastric cancer originates in the glandular epithelium of the stomach. In gastric cancer, CBX2 is capable of forming condensates that concentrate DNA and nucleosomes.^58^ Deletion of CBX2 blocks the YAP/β‐catenin pathway and inhibits the tumor cell proliferation, migration, and invasion.^211^ YAP mRNA and protein levels are upregulated in gastric cancer. Reintroduction of YAP in YAP‐mutated gastric cancer cell line MKN45 can promote its growth as subcutaneous tumors.^176^

Colorectal cancer originates in the mucosal epithelium of the colon and rectum. Concentrations of the super elongation complex component, ENL, were associated with colorectal cancer‐specific mortality.^222^ Recent studies have shown that the condensates formed by ENL can isolate and concentrate positive TF b in inactive HEXIM1 and activate transcription processes.^83^ BRD4 is strongly enriched at TERT promoter in colon cancer cells.^186^ Splicing factor proline‐ and glutamine‐rich loss can reduce the proliferation of colorectal cancer cells driven by BRAFV600E kinase and specifically induce S‐phase arrest and apoptosis.^187^ High level of YAP expression is also a factor for poor prognosis and is associated with cetuximab resistance.^188^

In ovarian cancer cells, DAXX interacts with promyelocytic leukemia protein and is localized to nuclear bodies PML in the subnuclear domain. This process enhances proliferation, colony formation, migration, and drug resistance in multiple ovarian cancer cell lines, whereas RNA interference with DAXX can reverse these processes.^189^ Besides, human ovarian cancer cell line treated with 17‐allylamino‐17‐demethoxygeldanamycin was accompanied by downregulation of HP1α expression.^190^

In addition, BRD4 recruits transcriptional regulatory complex to acetylated chromatin, and its inhibitors are therapeutic for myeloma.^197^, ^198^ In cervical cancer, NSD3 and JMJD6 are recruited into regulatory genes in a BRD4‐dependent manner to regulate transcriptional activity.^191^ Gαq can promote YAP‐dependent growth of uveal melanoma cells.^200^ Verteporfin, a YAP inhibitor, blocks tumor growth in uveal melanoma cells containing the Gq/11 mutations.^199^ Pancreatic‐specific knockout of YAP improves tumor progression in mouse model of pancreatic cancer.^223^ YAP/TAZ signaling pathway is also significantly changed in skin cancer^224^ and malignant pleural mesothelioma tumors, and the inhibitors of YAP/TAZ signaling pathway can effectively reverse the phenotype of the diseases.^185^ Besides, speckle‐type pox virus and zinc finger protein act as substrate connectors for cullin 3‐based E3 ligase. It is capable of target proteins for ubiquitination and subsequent proteasome degradation, and its abnormality may be involved in the occurrence and progression of human kidney cancer.^204^

### 5.2 Condensates are critical for immune disease

In innate immunity, the dsDNA sensor cGAS can form condensates with dsDNA or RNA to activate downstream signaling pathways. Abnormal cGAS activity can lead to diseases such as Aicardi‐Goutieres syndrome.^148^ G3BP1 is an important antiviral protein that is essential for SG assembly in innate immunity, which activates innate immune responses through transcription of NF‐κB and JNK.^161^ IKBKG can form condensates with polybiquitin chains.^113^ A decrease in condensates of IKBKG due to mutations leads to human immunodeficiency, while an increase leads to inflammatory diseases.^183^ SIRT1 agonists can inhibit the hyperacetylation of IRF3/IRF7 in DNA‐binding domain, promote the formation of condensates, activate innate immunity, and ultimately reduce viral load and mortality in mice.^151^ Herpes simplex virus is the cause of various herpes diseases including cold sores, interstitial keratitis, and encephalitis. Nuclear bodies nuclear domain 10 are able to cluster on viral DNA and restrict the expression of viral genes.^155^ NLRP6 can form condensates with dsRNA to induce inflammasome activation and interferon production to perform host defense functions.^164^ Varicella‐Zoster virus tegument protein ORF9 can bind to cGAS and form condensates with DNA, which inhibiting cGAS to produce cGAMP, and eventually causing chickenpox and shingles.^153^ Notably, herpesvirus‐coated membrane proteins ORF52 and VP22 undergo phase separation with DNA in vitro and in cells, which effectively disrupt preformed condensates of cGAS–DNA.^154^ The respiratory syncytial virus nucleocapsid protein can form condensates with p65 and block the innate immune interferon signal pathway.^166^ SARS‐CoV‐2 nucleocapsid RNA and protein also form condensates that allow efficient transcription of viral RNA, which can be interfered by small molecules or biologics.^158^ The condensates of STING were able to constrained TBK1 to prevent excessive activation of innate immunity, while microtubule inhibitors could hinder the generation of these condensates and increase the production of type I interferon in DNA virus‐infected cells.^137^ Moreover, the condensates formed by WASP, SRSF2, RNA, and Pol II are able to control RNA transcription and splicing, and defects in condensate formation lead to Wiskott‐Aldrich syndrome.^184^

In adaptive immunity, GRB2 and SOS1 can form condensates, which bind to T cell adaptor protein LAT and promote thymus cell development.^141^ PLCγ1 is also able to directly cross‐link LAT through its two SH2 domains to form condensates that protect LAT from dephosphorylation by CD45 and promote LAT‐dependent ERK activation and SLP76 phosphorylation.^142^ In addition, effective B cell activation requires condensates formed by the proline‐rich motif of SLP65, the nine SH3 domains of trimeric CIN85, and the lipid vesicle.^146^

### 5.3 Condensates in amyotrophic lateral sclerosis, Kabuki syndrome, and heart disease

Amplification of short nucleotide repeats contributes to the formation of condensates and can cause several neurological and neuromuscular diseases. *DMPK* forms condensates and produces amyotrophic lateral sclerosis.^179^ FUS contains intrinsic disordered domains is able to form condensates at sites of DNA damage and in stressed cytoplasm and associated with the neurodegenerative disease.^12^ Condensates formed by TAF15/hnRNP A1/hnRNP A2 are important drivers of amyotrophic lateral sclerosis.^172^ In addition, the altered condensates of TDP‐43 can selectively modify its RNA‐regulatory network, further affecting the course of amyotrophic lateral sclerosis.^180^

Kabuki syndrome patients have characteristic facial features, mild bone abnormalities, intellectual impairment, and postpartum growth defects. MLL4 regulates chromatin compartmentalization by forming condensates, and haploinsufficiency of MLL4 leads to changes in nuclear architecture in Kabuki syndrome.^181^ Phosphatidylserine and phosphatidylethanolamine can undergo phase separation in the presence of calcium ions. These condensates are associated with irreversible destruction of sarcolemma, ischaemia, ischaemia and reperfusion, and the calcium paradox.^173^ Furthermore, SHP2 mutants form condensates that recruit and activate wild‐type SHP2 to promote MAPK activation, which results in developmental disorders in humans.^182^

Together, biomolecular condensates can be therapeutic targets for diseases such as cancer, amyotrophic lateral sclerosis, kabuki syndrome, and heart and immune diseases. These evidence make condensates an essential candidate for therapeutic intervention. Therefore, the regulation of condensates is becoming a timely and exciting challenge. Related research will open up more possibilities for future drug discovery.

## 6 REGULATORY TARGETS AND METHODS OF BIOMOLECULAR CONDENSATES

### 6.1 Regulatory targets of biomolecular condensates

Emerging understanding of the mechanisms of condensate formation and function provides novel therapeutic opportunities. Among the components involved in the condensates, many of them were found to be able to serve as regulatory targets. In this section, we comprehensively discussed the latest findings of regulatory targets of biomolecular condensates. These targets include nucleic acid, amino acid, protein repeat domain, intrinsically disordered region (IDR), macromolecule, ATP, and pH.

#### 6.1.1 Nucleic acid serves as a key component of condensates

**FIGURE 6:**
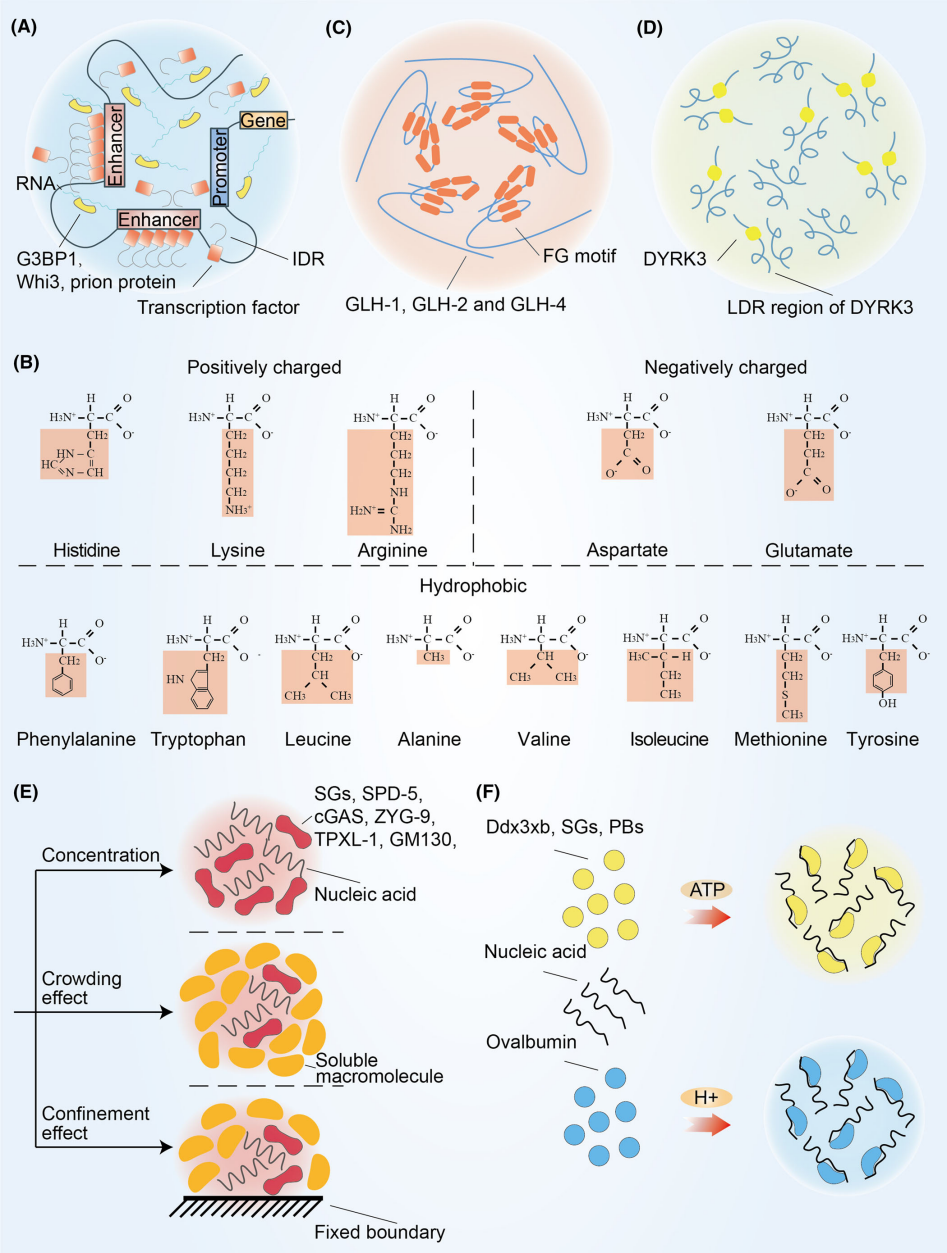
Regulatory targets of biomolecular condensates. The six regulatory targets of biomolecular condensates: (A) nucleic acids are essential for the formation of condensates such as G3BP1, Whi3, and prion protein; (B) amino acids such as positively charged amino acids, negatively charged amino acids and hydrophobic amino acids are involved in the formation of condensates; (C) protein repeat domain such as the FG motif of GLH‐1, GLH‐2, and GLH‐4 is critical for condensates stability; (D) intrinsically disordered region is able to regulate the formation of condensates such as DYRK3; (E) concentration, crowding effect and edge effect can regulate the formation of condensates; (F) ATP is involved in the formation of condensates such as Ddx3xb, SGs, and PBs; pH is related to condensate formation such as Ovalbumin. The red area in (B) represents the R group of amino acid. IDR, intrinsically disordered region; PrP, prion protein; SPD‐5, spindle‐defective protein 5; cGAS, cyclic GMP–AMP synthase.

In many cases, nucleic acid functions to facilitate phase separation (Figure 6A). DNA binds to heterochromatin protein 1α and promotes phase separation.^49^ RNA binds to G3BP1 to promote the formation of condensates then regulate the translation process.^225^ There are two highly conserved polybasic regions in the N‐terminal of PrP fragment, which have charge complementarity with nucleic acid.^18^ In the presence of 150 mM NaCl, the addition of crude tRNA promotes PrP phase separation, which further turn into solid‐like condensates.^18^ However, during phase separation of prion‐like RBPs, a high RNA:protein ratio inhibits formation of condensates.^226^ Intriguingly, RNA not only contributes to the formation of condensates but also is able to regulate itself by binding to condensates. The polyglutamine (polyQ) protein Whi3 generates different condensates depending on the RNA sequence further induce RNA conformational changes.^227^

#### 6.1.2 Amino acid affects the formation of condensates

Amino acid mutations or modifications can modulate phase separation (Figure 6B). Phosphorylation of serine 149 of G3BP inhibits the formation of SGs.^228^ The adaptor protein Nck promotes condensate formation through three SRC homology 3 domains that bind to multiple proline‐rich fragments in N‐WASP, and an SH2 domain binds to multiple phosphotyrosine sites in Nephrin.^229^ Three evenly distributed tryptophan‐binding pockets in Argonaute2 and glycine/tryptophan‐rich domain in TNRC6B produce condensates.^230^ Moreover, different phosphorylation patterns in serine/threonine of fragile X mental retardation protein and tyrosine of CAPRIN1 are associated with phase separation.^231^

#### 6.1.3 Protein repeat domain contribute to condensate formation

Protein repeat domain is critical for the formation of biomolecular condensates (Figure 6C). A lot of evidence showed that mutated protein repeats change the solubility of proteins and contribute to a variety of developmental and neurodegenerative diseases.^232^ It has been reported that the VASA‐related P granule proteins GLH‐1, GLH‐2, and GLH‐4 contain nuclear pore complex‐like FG repeat domains in *C. elegans*.^51^

#### 6.1.4 IDR is required for proteins to form condensates

Intrinsically disordered proteins refer to functional proteins that cannot fold into a clear spatial structure (Figure 6D), which typically have the IDR.^233^ Proteins with IDRs are also involved in multivalence protein interactions and tend to form condensates.^5^ The N‐terminus of dual‐specificity kinase DYRK3 form SGs through its IDR and kinase activity.^234^ IDR of FUS promote condensate formation when DNA damage or cytoplasmic stress.^124^ Moreover, CsoS2, Ddx4, NPM1, and Xvelo also promote phase separation through homotypic interactions of its IDR.^15^, ^31^, ^106^, ^235^

#### 6.1.5 Macromolecule crowding and confinement promote the assembly of condensates

It has been reported that the addition of organic additives and salts increases crowding and confinement effects in solution and further influence the formation of condensates (Figure 6E).^236^ Crowding effect refers to the volume repulsion effect of one soluble macromolecule on another soluble macromolecule, and confinement effect refers to effect of the fixed boundary on solubility of the macromolecule. These effects promote the assembly of SPD‐5, cGAS, microtubule polymerase ZYG‐9, and microtubule stabilizing protein TPXL‐1 into condensates.^14^, ^45^ Moreover, although the Golgi protein GM130 is not an inherently disordered protein, its overexpression still enables the formation of droplets.^237^ Notably, molecular chaperones can partially counteract these effects by allowing the protein to fold within the chaperone cavity and releasing the native‐like protein.^238^ Protein disulfide isomerase, a protein folding catalyst acts as a molecular chaperone, prevent the accumulation of lysozyme in the crowded state.^239^

#### 6.1.6 ATP and pH regulate the formation of condensates

Recently, the dynamic changes of ATP and pH related to cellular respiration have been discovered in process of phase separation (Figure 6F). ATP participated in the assembly of SGs and viscosity of the nucleolus.^24^ Shi et al.^240^ revealed that ATP promote Ddx3xb undergo liquid–liquid phase separation through its N‐terminal IDR. Besides, either ATP or the DEAD‐box ATPase Dhh1 modulate the formation of PBs.^241^ Changes in pH also affect the size and solubility of condensates.^242^ A lower pH lead to a decrease in cytoplasmic fluidity and inhibits phase separation of ovalbumin.^243^

Collectively, we summarize the current regulatory targets of biomolecular condensates, which are capable of acting as drivers and disruptors of condensate formation and function. More importantly, condensates are considered to be an emerging explanation for a large number of previously unknown phenomena, and further exploration is needed to explore more available regulatory targets for regulating condensates to interfere with various physiological and pathological functions.

### 6.2 Regulatory methods of biomolecular condensates

**FIGURE 7:**
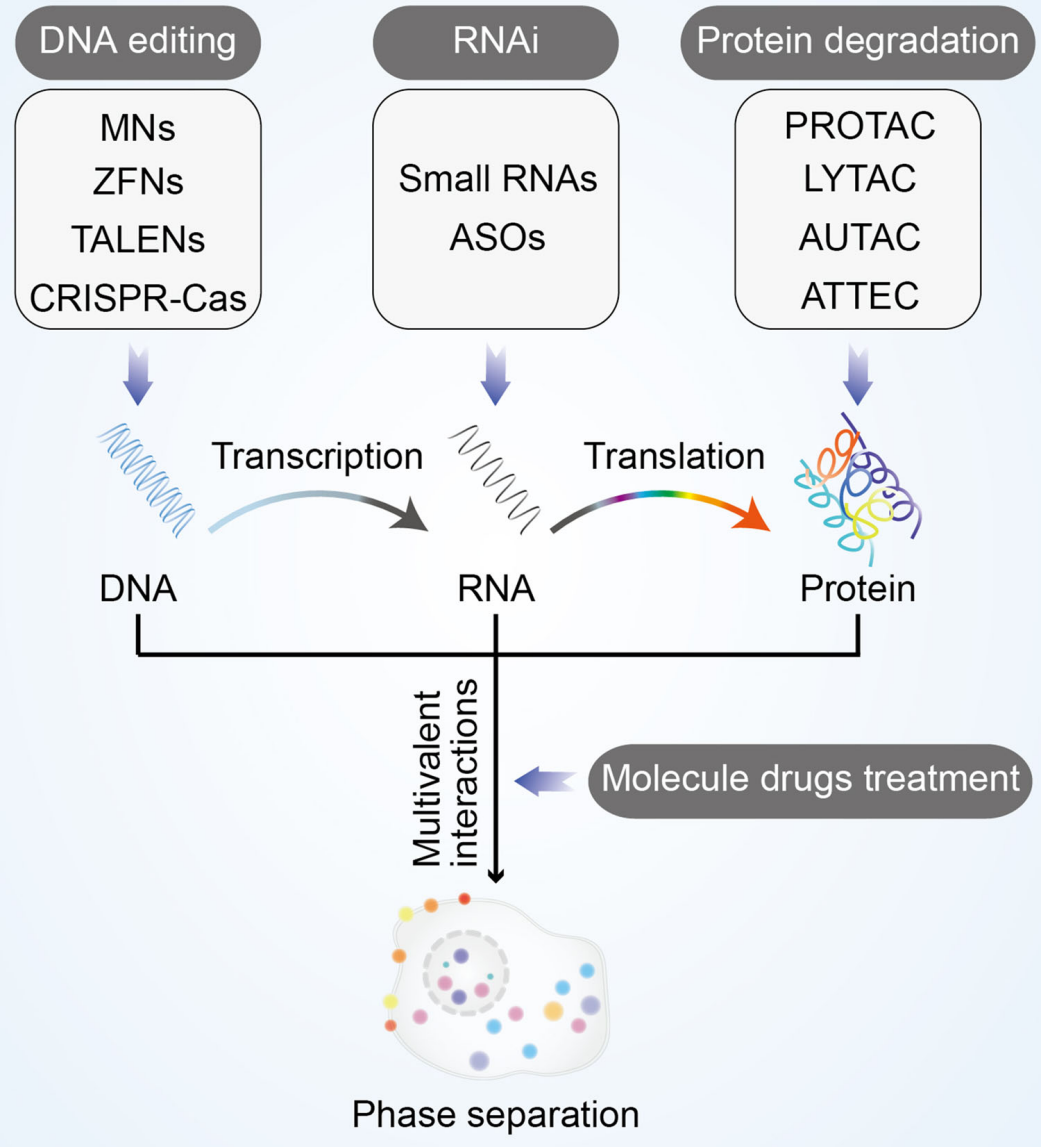
Regulatory methods of biomolecular condensates. The four regulatory methods of biomolecular condensates. DNA editing, RNAi, protein degradation, and molecule drug treatment target different processes of condensate formation. MNs, meganucleases; ZFNs, zinc‐finger nucleases; TALENs, transcription activator‐like effector nucleases; CRISPR/Cas, clustered regularly interspaced short palindromic repeats (CRISPR)/CRISPR‐associated endonuclease (Cas); ASOs, antisense oligonucleotides; PROTAC, proteolysis targeting chimera; LYTAC, lysosome‐targeting chimera; AUTAC, autophagy‐targeting chimera; ATTEC, autophagosome‐tethering compound.

In order to regulate the formation of biomolecular condensates, here are some methods that can be used against the above six targets. Modulating the formation of these condensates provides potential strategy for treatment of diseases. In this section, we comprehensively discussed the latest findings of available regulatory methods of biomolecular condensates (Figure 7). These methods include DNA editing, RNA interference, protein degradation, and molecule drugs treatment.

#### 6.2.1 Editing DNA involved in condensate formation

DNA editing relies on DNA recognition elements and a variety of endonucleases to introduce mutations into genes, which interfere the formation of condensates at the DNA stage.^244^ There are four types of DNA editing systems commonly used: meganuclease, zinc‐finger nuclease, transcription activator‐like effector nuclease, and CRISPR‐Cas system. Meganucleases are also known as homing endonucleases, which are a class of highly sequence‐specific and efficient enzymes found in yeast.^245^ Meganucleases system can specifically recognize DNA sequences ranging in length from 14 to 40 base pairs, then cleave homologous alleles without introns to generate double‐strand breaks and stimulate gene repair processes for homing.^246^ The zinc‐finger nuclease system relies on zinc finger proteins and endonucleases. Zinc finger proteins can bind DNA and recognize 9 bp DNA through their Cys2‐His2 fingers, and endonucleases can induce double‐strand breaks at specific positions in DNA, further achieving the purpose of gene deletion or addition through nonhomologous end joining or homology‐directed repair pathways.^247^ The transcription activator‐like effector nuclease system is similar as the zinc‐finger nuclease system, which also requires the fusion of DNA‐binding elements to the nonspecific FokI nuclease. The difference between two systems is that the DNA‐binding elements in the transcription activator‐like effector nuclease system are derived from *Xanthomonas* transcription activator‐like effectors, which contain a highly conserved repeat sequence that recognizes specific DNA sequence.^248^ The CRISPR/Cas system was originally discovered in bacteria and shows an antiviral function by cleaving nucleic acids that invade cells, which is currently the most used gene editing system.^249^ This system can cut DNA sequences via two pathways: one relies on multiprotein complexes, and the other relies on a single Cas protein. Notably, these systems have the disadvantage of off‐target editing.^250^, ^251^, ^252^

Recently, Shin et al. recently reported CasDrop, a novel optogenetic technology developed based on DNA editing, regulate phase separation of inherently disordered proteins.^98^ Intriguingly, the presence or absence of the specific nucleic acid can be further determined by DNA editing‐based phase separation. The 60 bp poly‐T DNA and poly‐l‐lysine promote phase separation and result in a turbid solution, whereas the addition of CRISPR/Cas12a or Cas13a cleaves specific DNA or RNA sequences and inhibits the formation of condensates.^253^

#### 6.2.2 Interfering RNA disrupts biomoleculars to form condensates

RNA interference (RNAi) modulate phase separation at the RNA stage. There are two types of RNAi used: small RNA and antisense oligonucleotides (ASOs).^254^ The small RNA can be divided into three categories: short interfering RNAs, repeat‐associated short interfering RNAs, and microRNAs. In the process of gene silencing, these RNAs rearrange in the RNP to form RNA‐induced silencing complexes, which further recognize complementary mRNA through its single‐stranded siRNA and induce mRNA degradation.^255^ ASOs are single strands of deoxynucleotides that usually 8–50 bp in length, which were found to have the ability to inhibit the translation of Rous sarcoma viral RNA in 1978.^256^ ASOs can bind to RNA to form a DNA–RNA complex, which enables RNA to be degraded by RNase H and ultimately inhibit protein expression.^257^

Recent studies have shown that the fluidity of PAR and their response to extracellular osmotic pressure can be reduced by small RNA.^258^ NONO‐TF E3 translocation renal cell carcinoma is a subtype of renal cell carcinoma.^259^ Small RNA blocks their phase separation and promotes GSK3β‐mediated degradation.^260^ Abnormal amplification sequences are toxic to cells and are sufficient to result in trinucleotide repeat expansion diseases such as Alzheimer's disease, Parkinson's disease, Huntington's disease, amyotrophic lateral sclerosis, and prion diseases.^261^ The RNA sequences of them contain a regular pattern of G and C nucleotides that are capable of intermolecular multivalent interactions that further lead to phase separation.^232^ Small RNA and 6×CTG ASOs induce RNase H‐catalyzed degradation of huntingtin mRNA and alleviate disease phenotype in the cerebrospinal fluid.^179^, ^262^ Moreover, mutations in SOD1 result in neurotoxicity and lead to amyotrophic lateral sclerosis.^263^ Therapies that directly deliver ASOs of SOD1 reduce the amount of mutant SOD1 in fibroblasts from amyotrophic lateral sclerosis patients and significantly slow disease progression.^264^

#### 6.2.3 Eliminate proteins to prevent condensate assembly

There are four types of protein degradation technologies commonly used: proteolysis targeting chimera, lysosome‐targeting chimaera, autophagy‐targeting chimera, and autophagosome‐tethering compound (ATTEC).^265^ The protein degradation function of the proteolysis targeting chimera relies on the UPS system in the cell that bind both the target protein and the ubiquitin ligase, resulting in the ubiquitination and further degradation of the target protein.^266^, ^267^ The lysosome‐targeting chimaera, autophagy‐targeting chimera, and ATTEC rely on lysosomal degradation system. The lysosomal degradation system needs lysosomes to degrade extracellular substances, plasma membrane proteins, cytosolic components, or organelles. There are two lysosomal degradation pathways in eukaryotic cells: the endosome–lysosomal pathway and the autophagy pathway. Specifically, the endosome–lysosomal pathway degrades endocytic substances and excess cellular components through a membrane binding process.^268^ In autophagy pathway, a part of the cytoplasm is engulfed by phagocytes to form autophagosomes, which are further fused with and degraded by lysosomes.^269^

These protein degradation methods can degrade polyQ proteins, such as mutant ATXN3, which can result in type III spinocerebellar ataxia disease. Li et al.^270^ reported a small‐molecule compounds interact with LC3 and mutant HTT but not with wild‐type HTT protein, reduce mutant HTT protein and improve phenotype in Huntington's disease. Intriguingly, nonprotein substrate molecules can also be degraded by ATTEC.^271^

#### 6.2.4 Efficient regulation of condensate formation by molecule drugs

Molecule drugs treatment can modulate phase separation effectively. 1,6‐Hexanediol inhibits phase separation by disrupting hydrophobic interactions.^272^ 1,6‐Hexanediol inhibits phase separation of architectural long noncoding RNA *NEAT1* and prevents assembly process of paraspeckles.^77^ Qiao et al.^273^ reported pinin, a protein localizes to desmosomes and nucleus, formed condensates with METTL3 then suppressed by 1,6‐hexanediol. Moreover, 1,6‐hexanediol prevents phase separation of SARS2‐NP, IDR‐rich scaffold protein NSP5, RNA chaperone NSP2, BRD4, and MED1 effectively.^90^, ^121^, ^274^ Furthermore, specific concentrations of 1,6‐hexanediol lead to nucleosome clutches larger and more evenly distributed in the cells, result in irreversible changes in chromatin.^275^

BRD4 and MED1 are two bromodomain‐containing molecules that modulate the formation of transcriptional condensates. The inhibitor JQ1 can bind to their bromodomain and prevent phase separation.^80^ MED1 also forms condensates with estrogen receptor alpha in an estrogen‐dependent manner.^94^ Tamoxifen is able to inhibit estrogen production and prevent the formation of the MED1 condensates.^80^ Besides, condensates formed by mediator and TFs are sensitive to transcriptional inhibitors.^93^ Ammonium acetate at 100 mM interferes with multivalent interactions in the 47×CAG RNA condensates and further leads to its degradation.^179^ Doxorubicin also blocks the formation of CAG RNA condensates in vitro.^179^

Together, DNA editing, RNAi, protein degradation, and molecule drugs treatment can regulate phase separation effectively. However, these methods still have off‐target effects with unpredictable and irreversible consequences and the possible impact needs to be carefully considered. The combination of biochemistry and biophysics will greatly benefit the development of new techniques. These technologies can further provide new opportunities for basic and clinical research in related diseases.

## 7 CONCLUDING REMARKS AND FUTURE PERSPECTIVES

In the past few years, significant progress has been made in the biomolecular condensates. In this review, we highlighted the importance in elucidating the regulation of biomolecular condensates. Amplification, transcription, translation, and posttranslational modification process at molecular scale, and transport and signal transduction at cellular scale process are all involved in condensates. Numerous diseases were caused by aberrant condensate formation. There are a range of regulatory targets and methods functions depend on the formation and function mechanism of condensates, which provides new opportunities for drug development and clinical treatment of diseases.

Biomolecular condensates are emerging as attractive new targets for drug discovery. Many proteins and nucleic acids of high therapeutic value all work in condensates. There have been a series of reports suggesting that condensates are druggable. A number of approved drugs such as cisplatin and tamoxifen that have been shown to form condensates that affect the concentration and activity of the drug.^80^ Drug‐like molecules that modulate the formation of condensates in a selective manner have been identified by screening.^121^, ^276^ Posttranslational modification has a strong regulatory effect on the formation and dissolution of condensates.^277^ There are a number of small molecules that have completed clinical trials have been able to target condensates (Table 3). It is tempting to speculate that many other potential drugs may exert their pharmacological benefit by modifying the condensates.

**TABLE 3:** Clinical trials on small molecules targeting biomolecular condensates.

Small molecules	Targets	Phase	Mechanisms	References
Avrainvillamide	NPM1	N/A	Avrainvillamide induced nuclear retention of NPM1 mutant protein, resulting in degradation of NPM1 mutant protein and nuclear export factor CRM1, and downregulated FLT3.	^278^
BAY 1892005	p53	N/A	BAY 1892005 interacts with p53 protein, resulting in the dissolution of condensates.	^99^
BAY 249716	p53	N/A	BAY 249716 interacts with p53 protein, resulting in the dissolution of condensates.	^99^
Cisplatin	MED1	Completed	Cisplatin preferentially modifies super‐enhancers, resulting in the dissolution of condensates.	^80^
Cyclopamine	IBs	N/A	Cyclopamine inhibits RSV replication by disrupting and hardening IB condensates.	^279^
EPI‐001	MED1	Completed	EPI‐001 blocks cofactor recruitment or DNA binding, impedes foci formation and thus androgen receptor transcriptional activity.	^280^
GSK‐626616	DYRK3	Completed	GSK‐626616 regulates organelle function, turnover, and maintenance of their spatial proximity and shape.	^281^
JQ1	MED1	N/A	JQ1 preferentially disrupts the transcription process.	^80^
Kanamycin	N‐Protein/ Viral RNA	Completed	Kanamycin reduced the size of condensates and the protein/RNA ratio in the reconstitution assay, and caused the relocalization of N‐protein to nucleus.	^282^
Leptomycin B	Nup98‐HoxA9	N/A	Leptomycin B resulting in the loss of chromatin binding of Nup98‐HoxA9 and Nup98‐HoxA9‐mediated activation of Hox genes.	^283^
Lipoamide	FUS	N/A	Lipamide reduced the formation of stress particles, promoted the regression of FUS within stressed cells, and reduced the accumulation of FUS in vitro and in vitro.	^284^
MG132	HSF1	N/A	MG132 prevents foci formation by modulating HSF1–DNA interaction.	^89^
Mitoxantrone	TDP‐43/FUS/ HNRNPA 2B1	Completed	Mitoxantrone prevents RNA‐dependent recruitment of ALS‐related RBP TDP‐43, FUS, and HNRNPA 2B1 into SGs.	^276^
	HSF1	N/A	Mitoxantrone can effectively prevent the formation of foci, and it can also dissolve the foci when applied after the formation of foci.	^89^
Oxaliplatin	FBL/NPM1	Completed	Oxaliplatin causes phase separation of nucleolus, leading to cell cycle arrest, Pol I‐mediated transcriptional shutdown, and ultimately cell death.	^285^
SHP099	SHP2	N/A	The SHP2 allosteric inhibitor SHP099 can attenuate the phase separation of SHP2 mutants, thereby enhancing the protein tyrosine phosphatase activity of SHP2.	^182^
SI‐2	SRC3	Completed	Using SI‐2 to target SRC‐3 or interfere with its interaction with NSD2 can overcome drug resistance in vitro and in vivo.	^74^
STA9090	HSF1	Completed	Disruption of protein homeostasis by STA9090 triggers chaperone induction and foci.	^89^
Tamoxifen	ER	Completed	Tamoxifen leads to eviction of ERα from the MED1 condensates.	^80^
THZ1	CDK7	N/A	THZ1 preferentially disrupt transcription processes.	^80^
	MED1	N/A	THZ1 inhibits tumor growth and reverses the drug resistance phenotype associated with hyperphosphorylated MED1 by blocking androgen receptor/MED1 corecruitment.	^203^

However, there are still several issues to be addressed in continued research. First, a large number of proteins should be further examined. Whether they function by forming condensates? Many proteins with known functions, especially TFs, may be involved in biological processes as condensates. As this hypothesis is tested, we will discover more ways to regulate these proteins. What molecular mechanisms are involved? Condensates are usually formed from more than one component, and the components do not necessarily interact directly with each other. Exploring the components in condensates will provide new targets for clinical treatment. Second, biomolecular condensates provide a large number of potential drug targets for treatment of diseases, such as aging diseases, and the phase transition of chemotherapeutic drugs in cancer treatment. What are the druggability of these targets? Preliminary evidence has shown that disrupt the assembly and disassembly of these condensates are important in health and disease. In order to fully develop approved drugs for clinical application, further validation of these molecular is needed. Third, how to develop more technologies based on the selective generation or degradation of condensates? Condensates are involved in a large number of biological functions, however, current regulatory methods still have side effects. There is an urgent need to integrate other disciplines to develop techniques for more effective intervention in condensate formation and function. Furthermore, the condensates are regulated by multivalent interactions and thermodynamic conditions and have relatively stable material properties. Therefore, it is attractive to develop an easily manipulated condensates system to selectively promote and inhibit the assembly and function of target molecules even if they do not contain IDR. Solving these problems may require drawing on disciplines such as biochemistry and biophysics. These issues must be met to provide novel therapies in the future.

## AUTHOR CONTRIBUTION

X. N. conceived and drafted the manuscript, drew the figures, and discussed the concepts of the manuscript. Lei. Z. conceived and drafted the manuscript, and discussed the concepts of the manuscript. Y.W. provided valuable discussion and funding. Z. Z. and B. W. discussed the concepts of the manuscript. Long. Z., F. Z., and J. L. provided valuable discussion and revised the manuscript. All authors have read and approved the final manuscript.

## CONFLICT OF INTEREST STATEMENT

The authors declare no conflict of interest.

## ETHICS STATEMENT

Not applicable.

## Data Availability

Not applicable.
